# Remote Work and Mental Health: A synthesis of risks, resilience factors, and Occupational Medicine’s evolving role

**DOI:** 10.1192/j.eurpsy.2026.11353

**Published:** 2026-07-31

**Authors:** C. Sridi, N. Ben Gadha, I. Kacem, N. Gannoun, J. Ben Khelifa, F. Chelly, N. Belhadj, A. Fki, M. Maoua

**Affiliations:** 1 Occupational Medicine Department, Sahloul University Hospital; 2 University of Sousse, Faculty of Medicine of Sousse; 3 Research Laboratory LR19SP03; 4 Occupational Medicine Department, Farhat Hached University Hospital; 5 Clinical Psychology Unit of Sahloul University Hospital, Sousse, Tunisia

## Abstract

**Introduction:**

The global shift to remote/hybrid work represents one of the most significant occupational transformations of the 21st century. While offering flexibility, this transition has introduced complex mental health challenges, including isolation, blurred work-life boundaries, and digital overload. Occupational physicians (OPs) are uniquely positioned to address these emerging risks, yet evidence-based frameworks for remote mental health support remain underdeveloped.

**Objectives:**

This review synthesizes current literature on remote work’s psychological impacts and identifies actionable strategies for OPs.

**Methods:**

A scoping review was conducted using PubMed, PsycINFO, and Web of Science (2020–2024). Search terms included “remote work,” “hybrid work,” “telework,” “mental health,” “occupational health,” “burnout,” “isolation,” and “digital wellbeing.” Inclusion criteria focused on peer-reviewed studies examining mental health outcomes in remote workers, with emphasis on longitudinal and cross-sectional research. Exclusion criteria covered non-occupational settings or non-mental health topics. Data extraction prioritized risk factors, protective mechanisms, and intervention efficacy.

**Results:**

Remote work exhibits a dual impact on mental health. Key risks include increased loneliness (reported by 67% of remote workers), elevated burnout due to extended screen time (average 3.1 additional hours/day), and reduced social support. Conversely, significant benefits emerge from autonomy (associated with 30% higher job satisfaction) and flexibility (linked to 25% lower stress in well-managed models). Critical protective factors include structured routines, supervisor support, and digital disconnection practices. OPs play a vital role in mitigating risks through early identification of digital fatigue, advocating for boundary-setting policies, and designing tailored mental health resources. Hybrid models show optimal outcomes when combining in-person social connection with remote autonomy.

**Conclusions:**

Remote work necessitates a paradigm shift in occupational mental health strategies. OPs must evolve from traditional workplace monitors to architects of healthy digital work ecosystems. Prioritizing three pillars, structured flexibility, intentional social connection, and proactive digital wellbeing protocols, can transform remote work from a risk factor into a sustainable model. Future directions include developing OP-led remote mental health screening tools and establishing evidence-based hybrid work standards.

**Disclosure of Interest:**

None Declared

